# Nonemergency colectomy for inflammatory bowel disease: the National Consultant Information Programme (NCIP) used to highlight colorectal surgery practice and outcomes in England 2018–2022 [Author-notes AN1]

**DOI:** 10.1308/rcsann.2025.0041

**Published:** 2025-06-19

**Authors:** A Galla, E Okoye, M Booth, L Young, A Taib, N Williams, J Hatt, C Maxwell-Armstrong

**Affiliations:** ^1^ University of Nottingham, UK; ^2^ Nottingham University Hospitals NHS Trust, UK; ^3^ NHS England, UK

**Keywords:** colectomies, UC, National Consultant Information Programme, IBD, Crohn’s

## Abstract

**Introduction:**

The National Consultant Information Programme (NCIP) tool provides a platform for NHS consultants to benchmark their practice and for trusts to have a purview of the range, variation and outcomes of surgical procedures. Surgery remains an effective strategy for the treatment of inflammatory bowel disease (IBD) despite advances in medical therapy. This study leverages the NCIP data to assess the current trends of colectomies for IBD and the impact of COVID-19.

**Methods:**

Pseudonymised data (demographics, number and types of colectomy per provider, resection approach and stoma formation) were collected from the NCIP for IBD Dashboard and analysed against outcomes such as readmission, length of hospital stay and mortality.

**Results:**

A total of 132 providers performed 3,907 colectomies for Crohn’s and 1,942 colectomies for ulcerative colitis (UC), with 76 (57.58%) performing more than 20 cases over the four-year period. More cases were performed laparoscopically, 55.46% and 61.17% for Crohn’s and UC, respectively. Transverse colectomy had the highest readmission rate (29%) for Crohn’s, whereas ileoanal anastomosis was highest at 29.37% for UC. Mortality rate was 0.23% and 0.82% for Crohn’s and UC, respectively.

Length of hospital stay was increased significantly in both Crohn’s disease and UC by open procedure and stoma creation.

**Conclusions:**

This study showed significant variation in practice across centres, with the volume of procedures performed in each centre being an influencing factor in the variation, especially when considering the incidence of stoma creation and surgical approach. This achieved the aim of the NCIP to keep a benchmark of standardisation across NHS practice.

## Introduction

Inflammatory bowel disease (IBD) is a chronic inflammatory disorder of the gastrointestinal tract that affects about 1.5 million people in North America, and 2.5–3.0 million in Europe.^1^

In the UK, studies suggest a prevalence of 725 per 100,000 in the UK, with an estimated number of about 500,000 people living with IBD.^2,3^ IBD requires complex strategies in its management and over the years there have been significant advances in medical therapy. Despite these advances, 20–30% of patients with ulcerative colitis (UC) require surgery for their disease at some point in their lives, whereas in Crohn’s disease (CD) the figure is as high as 50–70%. Nonemergency colectomies in particular are pivotal procedures aimed at alleviating symptoms and reducing the risks of complications in patients with refractory IBD.^4,5^

The National Consultant Information Programme (NCIP) is a free data platform that allows consultants to review their outcome data with the aim of improving clinical quality and patient safety. NCIP was first created in 2018 as part of the NHS’ Getting It Right First Time (GIRFT) programme, which itself is a national programme designed to improve the treatment and care of patients through an in-depth review of services, benchmarking and presenting a data-driven evidence base to support change and reduce unwarranted variation. NCIP provides an individual source of robust, nationally benchmarked data, which is updated every three months. Through its online portal, consultants can assess their own clinical practice activity and performance against local and national data. Currently, NCIP can be used for General surgery: upper gastrointestinal (GI) and lower GI, gynaecology, neurosurgery, orthopaedic surgery, paediatric surgery, spinal surgery, urology and vascular surgery, and with more specialties set to join.^6^ Data in the NCIP are from the English National Health Service Hospital Episode Statistics and the databases of the Office of National Statistics, from which mortality and demographic data are obtained. The portal contains diagnostic, demographic and procedural data for all inpatient stays, though it is important to note that NCIP in the main applies only to nonemergency admissions. Diagnostic coding is based on ICD-10 and procedures OPCS-4 (Office of Procedural Censuses and Surveys Classification of Interventions and Procedures).

This study uses NCIP data to explore the trends of nonemergency colectomies performed for IBD across the UK from April 2018 to March 2022. We aimed to assess variations in surgical techniques, evaluated patient outcomes and wished to document the impact of the COVID-19 pandemic on nonemergency procedures.

## Methods

This retrospective study is a quality improvement project of analysed anonymised patient and surgeon outcome data available on the NCIP portal from NHS England centres.

### Inclusion and exclusion criteria

Data were collected from the NCIP colectomy for IBD Dashboard and included all nonemergency colectomies from April 2018 to March 2022. Emergency or acute cases were excluded.

Demographic information collected on patients included age on admission, sex and ethnicity. Pseudonymised data on healthcare providers were collected, including the number and types of resection/procedure performed per provider, the resection approach, i.e. open, laparoscopic or robotic, and frequency of stoma formation.

The collected data also related to the period around the COVID-19 pandemic. Nonemergency colectomies performed between April 2018 and March 2020 were termed pre-COVID-19, while those performed between April 2020 and March 2022 were termed during COVID-19 pandemic.

### Dependent variables

Length of hospital stay (LOS), readmission and mortality rates were the main dependent variables used to compare outcomes of IBD nonemergency colectomies across NHS England. These outcomes were compared against patient demographics, type of resection, anastomosis, presence of stoma, surgical approach (open versus laparoscopic versus robotic) and provider caseload.

### Statistical analysis

Pseudonymised data were exported into a Microsoft Excel (version 16.0) spreadsheet and extracted appropriately to observe relevant trends in the data.^7^

Pearson chi-square test, odds ratio, one-sample *t*-test, independent samples *t*-test and paired *t*-tests were used to determine the relationship between variables and their statistical significance; *p* values <0.05 were considered statistically significant. Statistical analysis was performed using IBM SPSS Statistics version 22.0.^8^

## Results

A total of 132 providers in England recorded 3,907 colectomies for CD while 129 providers recorded 1,942 colectomies for UC over the data period. A summary of the key demographic and patient outcome metrics is presented in Table 1.

**Table 1:** Background data for total IBD patient cohort between April 2018–March 2022

	Crohn’s disease	UC
Total number of healthcare providers	132	129
Total number of cases	3,907	1,942
Mean number of cases per provider	29.6 (SD=24.2)	15.0 (SD=13.5)
Male (%), Female (%)	44.2%, 55.8%	39.6%, 60.4%
Frequency of stoma creation	32.9%	87.3%
Readmission within 30 days	17.3%	21.9%
Mortality within 90 days	0.23%	0.82%

SD = standard deviation; UC = ulcerative colitis

The mean number of Crohn’s colectomies performed per healthcare provider was 24 (1–129 cases), with 76 providers (57.58%) performing more than 20 cases over the four-year timeframe. The average number of UC colectomies performed per healthcare provider was 11 (1–81 cases), with 34 providers (26.36%) performing more than 20 cases over the four-year timeframe. The percentage of NHS cases performed by independent healthcare providers accounted for a small proportion of Crohn’s (2.33%) and UC (1.18%) cases.

**Figure 1:**
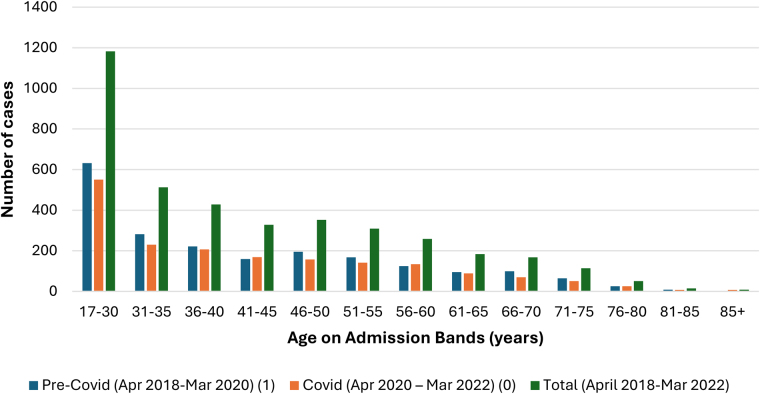
Frequency of Crohn’s cases according to the age of patient on admission and the impact COVID-19 pandemic had on case frequency.

**Figure 2:**
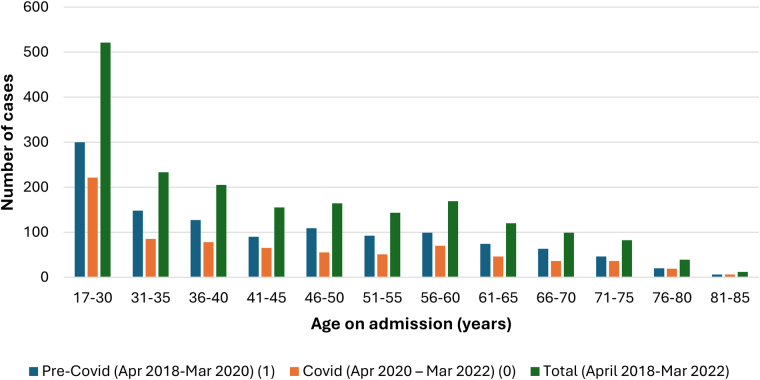
Frequency of UC cases according to the age of patient on admission and the impact COVID-19 pandemic had on case frequency. UC = ulcerative colitis

Figure 1 for Crohn’s and Figure 2 for UC demonstrate the age distribution of the patients, showing the total numbers per each age group, pre-COVID and during the COVID pandemic, in which most of the patients fall into the 17- to 30-year-old bracket and with a general reduction in case numbers as age increased. This group made up 30% of Crohn’s and 27% of UC cases. Over 80% of patients in both Crohn’s and UC were of white ethnicity.

### Procedure subgroups and surgical approach

**Figure 3:**
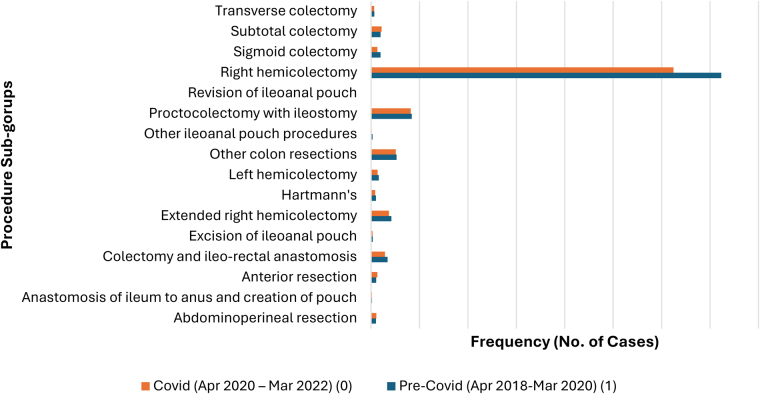
Bar graph illustrating frequency of Crohn’s cases (*N*=3,907)—Pre-COVID-19 and during COVID-19—categorised by procedure subgroups. Decreased observed in frequency of cases for the three most common procedure subgroups: right hemicolectomy, proctocolectomy with ileostomy and other colon resections. Minimal number of cases in other procedure subgroups, thus this is not analysed further in this project.

**Figure 4:**
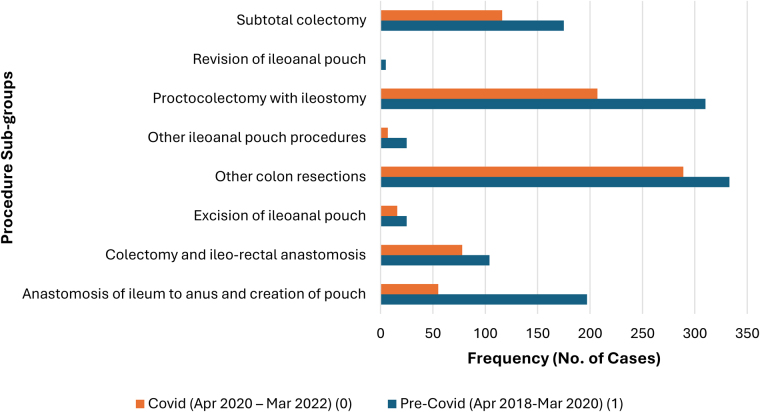
Bar graph illustrating frequency of UC cases (*N*=1,942)—Pre-COVID-19 and during COVID-19—categorised by procedure subgroups. Decreased observed in frequency of cases all procedure subgroups, notably for the three most common procedure subgroups: other colon resections, proctocolectomy with ileostomy and subtotal colectomy. UC = ulcerative colitis

Figures 3 and 4 show several procedure subgroups for Crohn’s and UC resections, respectively. The most frequent resection for CD was right hemicolectomy (68.98%), followed by proctocolectomy with ileostomy (8.5%). For UC, most cases performed were classified as other ‘colon’ resections (32%), followed by proctocolectomy with ileostomy (27%) and subtotal colectomy (15%). Colectomy and ileorectal anastomosis (IRA) accounted for 9% of cases. Cases involving pouch surgery were subdivided into pouch formation (13%), pouch excision (2%) and other (1.6%).

**Figure 5:**
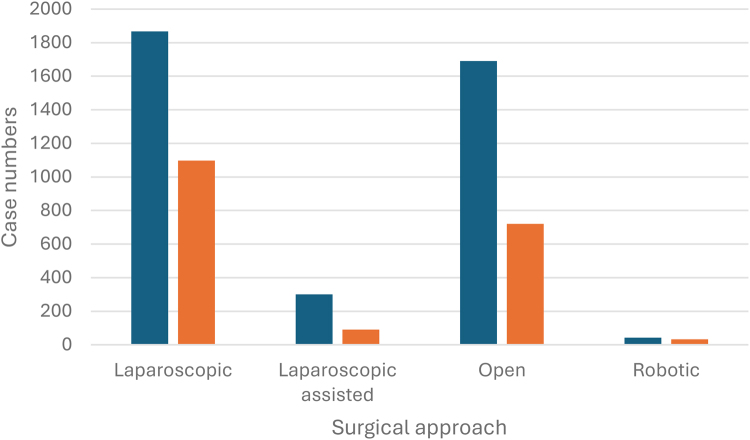
Case frequency of Crohn’s (blue) and UC (orange) according to surgical approach from March 2018 to April 2022. UC = ulcerative colitis

As shown in Figure 5, over half of Crohn’s cases were performed laparoscopically or laparoscopically assisted: 2.167 cases (55.5%). Open surgery was performed in 1,690 cases (43%), whereas robotic surgery was performed in 50 cases (1.2%).

As also shown in Figure 5, most UC cases were performed laparoscopically or laparoscopically assisted; 1,188 cases (61.2%). Open surgery was performed in 721 cases (37%), with robotic surgery performed in 33 cases (1.7%).

### Readmission rates and mortality

For Crohn’s colectomy cases, the overall readmission rate within 30 days was 17.33%. The highest percentage of readmission was in those who underwent a transverse colectomy (29%). Mortality within 90 days was 0.23% and highest in those who underwent a left hemicolectomy. In UC colectomy cases, the overall readmission rate within 30 days was 21.94% and highest in those patients who underwent anastomosis of the ileum to anus and creation of pouch (29.37%). The average mortality rate within 90 days was 0.82%, with the highest rates in those undergoing a colectomy and ileo-rectal anastomosis. There was no significant impact of stoma creation on the rate of readmission or mortality.

### Length of stay

Tables 2 and 3 show the effect of the surgical approach on LOS in Crohn’s and UC cases, respectively. We noted that the overall mean LOS for Crohn’s resections was 9.01 days (standard deviation (SD)=9.71) with 95% confidence interval (CI) (8.70, 9.32) and for UC cases, it was 10.39 days (SD=12.90) with 95% CI (9.82, 10.96), with most cases having a lower LOS. There were still a few cases with comparatively higher LOS, which could be due to individual factors and/or complications.

**Table 2:** Approach subgroups in Crohn’s resections and effect on mean LOS; *p*<0.001

Approach subgroups	*N*	Mean LOS (days)	SD	SEM	95% CI	
					Lower	Upper
Open	1,690	11.13	12.40	0.30	10.54	11.73
Laparoscopic	1,866	7.30	6.68	0.15	7.00	7.61
Laparoscopic assisted	301	7.81	5.85	0.34	7.15	8.47
Robotic	50	7.72	4.94	0.70	6.35	9.09
Overall	3,907	9.01	9.71	0.16	8.70	9.31

CI = confidence interval; LOS = length of hospital stay; SEM = standard error of the mean; SD = standard deviation

**Table 3:** Approach subgroups in UC resections and effect on mean LOS; *p*<0.001

Approach subgroups	*N*	Mean LOS (days)	SD	SEM	95% CI	
					Lower	Upper
Open	721	12.69	12.40	0.70	11.32	14.05
Laparoscopic	1,098	9.30	10.31	0.31	8.69	9.91
Laparoscopic assisted	90	10.00	10.25	0.98	8.06	11.94
Robotic	33	8.48	10.31	0.93	6.66	10.31
Overall	1,942	10.39	12.90	0.29	9.82	10.96

CI = confidence interval; LOS = length of hospital stay; SEM = standard error of the mean; SD = standard deviation; UC = ulcerative colitis

For Crohn’s cases, the longest mean LOS (11.13 days (SD=12.40)) was associated with open procedures, whereas the shortest mean LOS (7.30 days (SD=6.68)) was seen in those having a laparoscopic procedure. For UC cases, the overall mean LOS was 10.39 days (SD=12.90). When subdivided into surgical approaches, the longest mean LOS (12.69 days (SD=14.05)) was associated with an open procedure, with the shortest mean LOS (8.48 days (SD=10.31)) in those cases performed robotically.

Tables 4 and 5 show the effect of stoma creation on LOS in Crohn’s and UC cases, respectively. In cases where a stoma was created, the mean LOS for Crohn’s resections with stoma formation was 13.04 days (SD=13.77), while Crohn’s resection without stoma formation had a mean LOS of 7.03 days (SD=5.97). Stoma creation increased LOS significantly, by 6.01 days ±0.40 with 95% CI (5.23, 6.80); *p*<0.001.

**Table 4:** Stoma creation and its impact on mean LOS (in days) for Crohn’s disease^†^

Stoma created within episode (no:0, yes:1)	LOS statistics (in days)					
	*N*	Mean	SD	SEM	95% CI	
					Lower	Upper
0.00	2,622	7.03	5.97	0.12	6.80	7.26
1.00	1,285	13.04	13.77	0.38	12.29	13.79
Overall	3,907	9.01	9.71	0.16	8.70	9.31

CI = confidence interval; LOS = length of hospital stay; SEM = standard error of the mean; SD = standard deviation ^†^Overall mean LOS was 9.01 days±0.16 with 95% CI (8.70, 9.32). Stoma creation led to a higher mean LOS (13.04±0.38 with 95% CI (12.29,13.79)). These data on stoma creation and its impact on mean LOS for Crohn’s are statistically significant; demonstrated through independent samples *t*-test for equality of means (*p*-value<0.001) which yielded a *p*-value <0.05.

**Table 5:** Stoma creation and its impact on mean LOS (in days) for UC^‡^

Stoma created within episode (no:0, yes:1)	LOS statistics (in days)					
	*N*	Mean	SD	SEM	95% CI	
					Lower	Upper
0.00	247	8.61	8.38	0.53	7.57	9.66
1.00	1,695	10.65	13.42	0.33	10.01	11.29
Overall	1,942	10.39	12.90	0.29	9.82	10.97

CI = confidence interval; LOS = length of hospital stay; SEM = standard error of the mean; SD = standard deviation; UC = ulcerative colitis ^‡^Overall mean LOS was 10.39 days±0.29 with 95% CI (9.82,10.96). Stoma creation led to a higher mean LOS (10.65±0.33 with 95% CI (10.01, 11.29)). These data on stoma creation and its impact on mean LOS for UC are statistically significant; demonstrated through independent samples *t*-test for equality of means (*p*-value<0.05) which yielded a *p*-value <0.05.

Similarly, for UC cases, stoma creation increased LOS significantly. The mean LOS in UC patients with a stoma was 10.65 days (SD=13.42), compared with 8.61 days (SD=8.38) in those without a stoma, an increase of 2.04 days ±0.88 with 95% CI (0.32, 3.76); *p*<0.05.

### Rate of stoma creation

**Figure 6:**
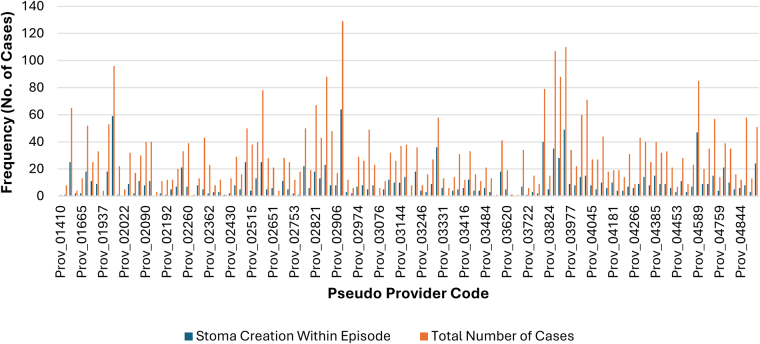
Bar graph illustrating frequency of stoma creation for Crohn’s compared with total frequency of Crohn’s cases performed by pseudonymised provider code. High variation in number of stomas created at each provider code; 76 units performing ≥20 cases (57.58%). Mean stoma rate was 31.73% in units performing ≥20 cases (range, 4.55–63.64%). These Crohn’s data on stoma creation and rate by pseudonymised provider code are statistically significant; demonstrated through Pearson chi-square test (*p*-value≤0.001) and the likelihood ratio (*p*-value <0.001), which both yielded *p*-values <0.05.

**Figure 7:**
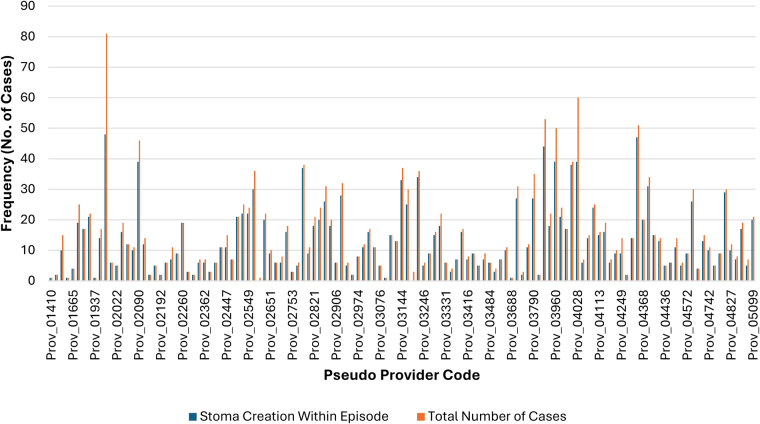
Bar graph illustrating frequency of stoma creation for UC compared with total frequency of UC cases performed by pseudonymised provider code. High variation in number of stomas created at each provider code. Additionally, the general trend observed is a high proportion of stomas created at most providers in proportion to total number of cases at the respective providers; 34 units performing ≥20 cases (26.36%). Mean stoma rate was 87.09% in units performing ≥20 cases (range, 59.26–100%). These UC data on stoma creation and rate by pseudonymised provider code are statistically significant; demonstrated through Pearson chi-square test (*p*-value ≤0.001) and the likelihood ratio (*p*-value <0.001), which both yielded *p*-values <0.05. UC = ulcerative colitis

Tables 6 and 7 showed the rate of stoma creation between individual units. A high rate of dispersion and variation was observed across the different providers. Of note on Figures 6 and 7, as shown on Figure 6, in Crohn’s cases, 76 out of 132 providers performed more than or equal to 20 cases per year. These providers had a combined mean stoma rate of 31.73% compared with a mean rate of 32.9% among all providers.

**Table 6:** Key descriptive statistics for frequency of stoma creation for cases of Crohn’s disease (*N*=3,907)^§^

*N*	132
Mean	9.73
SEM	0.97
Median	7.00
Mode	5.00
SD	11.10
Variance	123.25
Range	64.00
Minimum	0.00
Maximum	64.00

CI = confidence interval; IQR = interquartile range; SEM = standard error of the mean; SD = standard deviation ^§^132 providers, mean stoma creation was 9.73 cases±0.97 with 95% CI (7.83, 11.63). Median of 7.00 cases and mode of 5.00 cases days observed. High SD (11.10) and variance (123.25) observed, suggesting high level of dispersion and variation. High value observed for range (64.00). Skewness, Kurtosis, IQR and quartiles not calculated as high level of dispersion and variation has been clearly established through key statistics in this table.

**Table 7:** Key descriptive statistics for frequency of stoma creation for cases of UC (*N*=1,942)^¶^

*N*	129
Mean	13.14
SEM	0.95
Median	10.00
Mode	6.00
SD	10.77
Variance	116.04
Range	48.00
Minimum	0.00
Maximum	48.00

CI = confidence interval; IQR = interquartile range; SEM = standard error of the mean; SD = standard deviation; UC = ulcerative colitis ^¶^129 providers, mean stoma creation was 13.14 cases±0.95 with 95% CI (11.28,15.00). Median of 10.00 cases and mode of 6.00 cases days observed. High SD (10.77) and variance (116.04) observed, suggesting high level of dispersion and variation. Relatively value observed for range (48.00). Skewness, Kurtosis, IQR and quartiles not calculated as high level of dispersion and variation has been clearly established through key statistics in this table.

### Impact of COVID-19 pandemic

**Figure 8:**
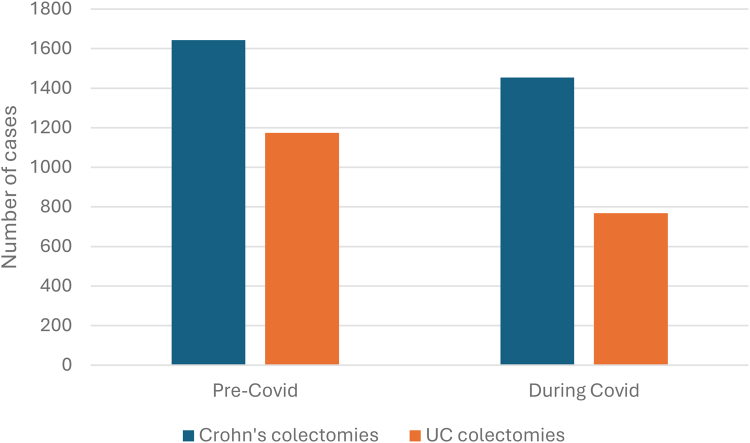
Total number of elective Crohn’s or UC colectomy cases occurring pre COVID-19 (April 2018–March 2020) and during the first two years of COVID-19 pandemic (April 2020–March 2022). UC = ulcerative colitis

Numbers for both Crohn’s and UC nonemergency colectomies fell during the COVID-19 pandemic. When comparing case frequency pre-COVID-19 and during COVID-19, there was a larger decrease observed for UC cases, where a 20.9% fall was seen (Figure 8). For UC cases, the decrease in frequency was seen in all procedure subgroups, most notably in three subgroups: anastomosis of the ileum to anus and creation of pouch, proctocolectomy with ileostomy and subtotal colectomy (Figure 4). Although the pandemic had no critical impact on the age of patients at admission, there was a decrease in cases performed in all age groups for both Crohn’s (Figure 1) and UC (Figure 2).

**Figure 9:**
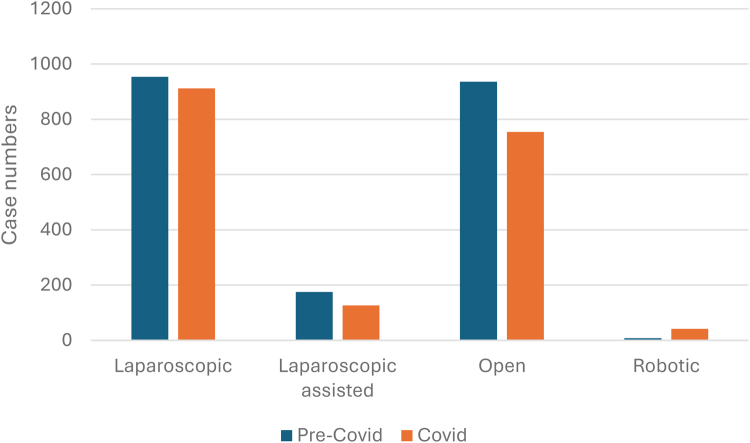
The impact of COVID-19 on surgical approach for elective Crohn’s colectomies. The decrease observed in frequency of cases according to surgical approach was statistically significant (*p*<0.001). Cross-tabulation of Crohn’s data by surgical approach (open, laparoscopic, laparoscopic assisted, robotic) and data period (pre-COVID-19, during COVID-19, total data period), illustrating frequency and percentage of cases (*N*=3,907). A decrease was observed in frequency and percentage of cases for all surgical approach subgroups except robotic (0.87% increase in percentage of total cases). These cross-tabulation data are statistically significant; demonstrated through Pearson chi-square test (*p*-value<0.001).

There were decreases in frequency seen in all forms of surgical approach for Crohn’s colectomies during COVID-19 except for robotic surgery. There were 36 more cases performed robotically during COVID-19 than pre-COVID-19; however, the total number of cases performed robotically accounted for only 1.07% of the total performed between April 2020 and March 2022 (Figure 9).

As with Crohn’s cases, all forms of surgical approach for UC colectomies decreased during the COVID-19 pandemic except robotic surgery. This approach was utilised in 13 more cases than pre COVID-19 but accounted for only 1.18% of cases performed between April 2020 and March 2022. It is important to note that the increase in the number of robotic procedures in this cohort is probably more related to the general advances in the use of robotic techniques rather than the effect of the pandemic itself.

When looking at the impact COVID-19 had on Crohn’s case frequency, there was a statistically significant fall in the average case numbers performed per unit from 14 to 10 (*p*<0.001). Similarly, the frequency of UC cases performed per healthcare provider fell following the start of the pandemic, with a statistically significant decrease in average number from 6 to 4 cases (*p*<0.001). While the general trend demonstrated higher frequency of UC cases being performed per unit before the start of the pandemic, several providers performed a higher frequency of cases during COVID-19 than pre-COVID-19. Additionally, select providers who performed no cases in the pre COVID-19 timeframe performed minimal case numbers during COVID-19.

## Discussion

This retrospective study provides insight into the surgical management of IBD in the UK. It provides a detailed analysis of the trends of nonemergency colectomies in patients with CD and UC over the period of study and demonstrates significant variation in certain outcomes. It also documents for the first time the impact of the COVID-19 pandemic on this particular group of patients.

The demographics showed a predominance of IBD cases among young patients aged 17 to 30 and a predominantly white ethnicity of more than 80%. This result is consistent with the known epidemiology of IBD, which shows that the disease is predominant in patients of Caucasian race and frequently manifests in a younger population.^9^

Right hemicolectomy was the most common colectomy performed for CD, which is consistent with the terminal ileum being the most affected in this disease.^10^ For UC, most cases (32%) were classified as ‘other colon resections’, which includes several procedures. Notably, completion colectomy was not named in this series and probably would have been coded as ‘other colon resections’ according to the OPCS 4 coding on the NCIP platform. The remainder were proctocolectomy with ileostomy, subtotal colectomy, colectomy and ileorectal anastomosis and pouch surgery, which were subdivided into pouch formation and pouch excision. Pouch formation accounted for 13% of this cohort.

Most cases were performed laparoscopically, with more than half of Crohn’s operations and about two-thirds of UC cases performed minimally invasively. This is consistent with current trends in colorectal resection.^11^ A small number of the cases were performed robotically. This showed an increasing trend during the COVID-19 pandemic. Advances in robotic colorectal surgery have largely been in patients with cancer, although it seems likely that going forward the proportion of IBD cases performed robotically will increase. It will be interesting to see whether this is at the expense of the laparoscopic group, or whether the proportion of cases performed using a minimal access approach increases overall.

Open surgery and stoma creation significantly increased LOS, with a difference of four days between the longest average LOS in open procedures and the shortest average LOS in laparoscopic procedures in both UC and CD, as would have been expected.

There was significant variation in the rate of stoma creation, temporary or permanent among individual units, with the main predictor being the volume of cases performed in each unit, as there was significantly less stoma creation in units that performed more than 20 cases. LOS increased more significantly in patients in whom a stoma was created than in those without. The presence of a stoma did not contribute any significant impact on re-admission or mortality. This is also consistent with previous studies stating that stoma creation significantly increases LOS due to required patient education and stoma care adaptation.^12^

There was considerable variation in the number of providers performing colectomies for CD and UC. Whereas some units handled only a few cases, others performed a substantially higher volume. This disparity indicates that a relatively small number of centres managed a large proportion of the surgical workload, probably reflecting differences in service structure, referral patterns and specialist expertise, and this influenced stoma rates (and thus LOS). As described above, thought needs to be given to centralisation of nonemergency IBD surgery to high-volume units, and potentially specialisation of surgeons in these units as has been proposed in rectal cancer.^13^ There is no place in the current NHS for a unit to be performing one nonemergency IBD procedure in a four-year period. The Association of Coloproctology of Great Britain and Ireland has established an accreditation process for ‘pouch’ surgery, which is to be commended.^14^ It is hoped that this will go some way to address the significant variation described, and consideration should also be given to extending it to other areas of IBD surgery.

There was a significant increase in overall 30-day readmission risk and mortality in UC cases compared with Crohn’s colectomies. Patients who underwent ileo-anal anastomosis with the creation of pouch had the highest readmission risk. This is consistent with the findings of Feuerstein *et al*, which showed ileal pouch-anal anastomosis as a single significant predictor of readmission within 30 days.^15^

Numbers for both Crohn’s and UC nonemergency colectomies fell during the COVID-19 pandemic. When comparing case frequency pre-COVID-19 and during COVID-19, there was a larger decrease observed for UC cases, where a 20.9% fall was seen. For UC cases, the decrease in frequency was seen in all procedure subgroups, but most notably anastomosis of the ileum to anus and creation of pouch, proctocolectomy with ileostomy and subtotal colectomy. These are clearly elective, and would have been classed as a lower priority than cancer resections. The data do however support the premise that a younger group of patients with IBD had significant delays in their care. What would have been of interest would have been to see whether the proportion of emergency colectomies for IBD increased during the pandemic, particularly in the subtotal colectomy group. There was certainly evidence that, during the COVID-19 pandemic, colorectal cancer presented with more advanced disease.^16^ This is, however, outside the remit of NCIP, where data are available only on patients undergoing nonemergency surgery.

## Conclusion

Our work shows that there is a significant variation in practice in the surgical treatment of patients with IBD. Some aspects, such as stoma creation, and choice of open versus minimally invasive procedures, remain largely influenced by the surgeon, with robotic surgery relatively under-represented during this time period.

There is a significant variation between centres in the volume of cases performed, with one unit performing only one case in four years. An important aim of NCIP and GIRFT is to highlight this degree of variation. Our data also highlight the significant impact the COVID-19 pandemic had on nonemergency IBD surgery.
